# (4-Benzyl-2-oxo-1,3-oxazolidin-5-yl)methyl methane­sulfonate

**DOI:** 10.1107/S1600536809055020

**Published:** 2010-01-09

**Authors:** Wilson Cunico, Claudia R. B. Gomes, Edward R. T. Tiekink, Walcimar T. Vellasco Junior, James L. Wardell, Solange M. S. V. Wardell

**Affiliations:** a Departamento de Química Orgânica, Universidade Federal de Pelotas (UFPel), Campus Universitário, s/n°, Caixa Postal 354, 96010-900 Pelotas, RS, Brazil; b Fundação Oswaldo Cruz, Instituto de Tecnologia em Fármacos–Farmanguinhos, R. Sizenando Nabuco 100, Manguinhos, 21041-250 Rio de Janeiro, RJ, Brazil; c Department of Chemistry, University of Malaya, 50603 Kuala Lumpur, Malaysia; d Centro de Desenvolvimento Tecnológico em Saúde (CDTS), Fundação Oswaldo Cruz (FIOCRUZ), Casa Amarela, Campus de Manguinhos, Av. Brasil 4365, 21040-900 Rio de Janeiro, RJ, Brazil; e CHEMSOL, 1 Harcourt Road, Aberdeen AB15 5NY, Scotland

## Abstract

The title compound, C_12_H_15_NO_5_S, features an approximately planar five-membered oxazolidin ring (r.m.s. deviation = 0.045 Å) with the peripheral benzyl and methyl methane­sulfonate residues lying to either side of the plane. In the crystal, N—H⋯O hydrogen bonds, involving one of the sulfur-bound oxo groups as acceptor, lead to the formation of supra­molecular chains along the *b* axis. These chains are reinforced by C—H⋯O contacts with the carbonyl O atom accepting three such inter­actions. The structure was refined as a racemic twin, with the major component being present 89% of the time.

## Related literature

For the use of 1,3-oxazolidin-2-ones as chiral auxiliaries in organic synthesis, see: Evans *et al.* (1981 ▶); Ager *et al.* (1996 ▶, 1997 ▶); Hinter­mann & Seebach (1998 ▶). For their biological activity, see: Poce *et al.* (2008 ▶); Brickner *et al.* (2008 ▶); Means *et al.* (2006 ▶); Kaiser *et al.* (2007 ▶); Clemmet & Markham (2000 ▶); Ebner *et al.* (2008 ▶); Negwer & Scharnow (2007 ▶); Mai *et al.* (2003 ▶). For background to their syntheses, see: Ochoa-Terán & Rivero (2008 ▶); Zappia *et al.* (2007 ▶).
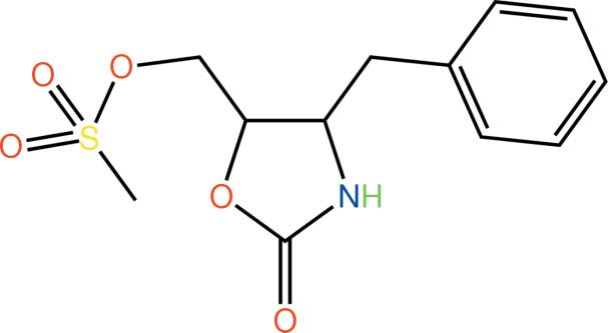


## Experimental

### 

#### Crystal data

C_12_H_15_NO_5_S
*M* *_r_* = 285.31
Monoclinic, 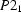

*a* = 8.7332 (5) Å
*b* = 5.8757 (3) Å
*c* = 12.9650 (7) Å
β = 103.317 (3)°
*V* = 647.39 (6) Å^3^
*Z* = 2
Mo *K*α radiation
μ = 0.27 mm^−1^
*T* = 120 K
0.26 × 0.08 × 0.02 mm

#### Data collection

Nonius KappaCCD area-detector diffractometer
Absorption correction: multi-scan (*SADABS*; Sheldrick, 2007 ▶) *T*_min_ = 0.700, *T*_max_ = 1.000
6748 measured reflections
2587 independent reflections
2266 reflections with *I* > 2σ(*I*)
*R*_int_ = 0.059

#### Refinement

*R*[*F* ^2^ > 2σ(*F* ^2^)] = 0.057
*wR*(*F* ^2^) = 0.123
*S* = 1.11
2587 reflections
177 parameters
2 restraints
H atoms treated by a mixture of independent and constrained refinement
Δρ_max_ = 0.33 e Å^−3^
Δρ_min_ = −0.36 e Å^−3^

### 

Data collection: *COLLECT* (Hooft, 1998 ▶); cell refinement: *DENZO* (Otwinowski & Minor, 1997 ▶) and *COLLECT*; data reduction: *DENZO* and *COLLECT*; program(s) used to solve structure: *SHELXS97* (Sheldrick, 2008 ▶); program(s) used to refine structure: *SHELXL97* (Sheldrick, 2008 ▶); molecular graphics: *ORTEP-3* (Farrugia, 1997 ▶) and *DIAMOND* (Brandenburg, 2006 ▶); software used to prepare material for publication: *publCIF* (Westrip, 2010 ▶).

## Supplementary Material

Crystal structure: contains datablocks global, I. DOI: 10.1107/S1600536809055020/hb5289sup1.cif

Structure factors: contains datablocks I. DOI: 10.1107/S1600536809055020/hb5289Isup2.hkl

Additional supplementary materials:  crystallographic information; 3D view; checkCIF report

## Figures and Tables

**Table 1:** Hydrogen-bond geometry (Å, °)

*D*—H⋯*A*	*D*—H	H⋯*A*	*D*⋯*A*	*D*—H⋯*A*
N1—H1*N*⋯O2^i^	0.88 (2)	2.28 (2)	3.113 (5)	160 (4)
C1—H1*B*⋯O5^ii^	0.98	2.51	3.428 (6)	155
C2—H2*A*⋯O5^ii^	0.99	2.32	3.214 (5)	150
C5—H5⋯O5^iii^	1.00	2.36	3.326 (6)	162
C6—H6*B*⋯O1^iv^	0.99	2.59	3.528 (6)	158

Symmetry codes: (i) 
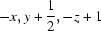
; (ii) 
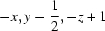
; (iii) 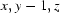; (iv) 
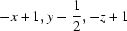
.

**Fig. 1.**
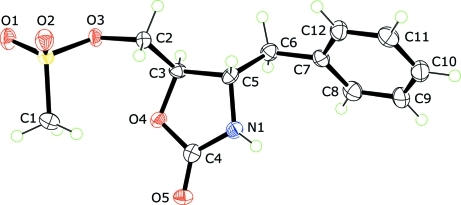
The molecular structure of (I) showing displacement ellipsoids at the 50% probability level.

**Fig. 2.**
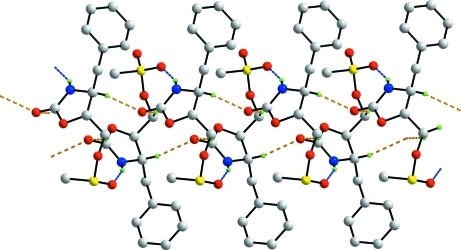
A view of the supramolecular chain in (I) mediated by N–H···O hydrogen bonding and C–H···O contacts, shown as blue and orange dashed lines, respectively. Hydrogen atoms not involved in intermolecular contacts sustaining the chain are omitted for reasons of clarity. Colour code: S, yellow; O, red; N, blue; C, grey; and H, green.

**Fig. 3.**
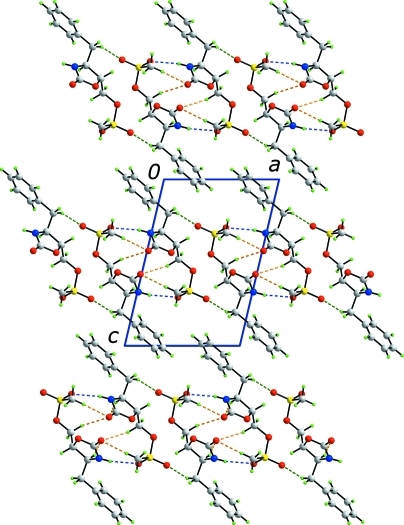
A view of the stacking of layers in (I) with partial the interdigitation of the benzene rings. The N–H···O hydrogen bonding and C–H···O contacts stabilizing the supramolecular chains are shown as blue and orange dashed lines, respectively. The C–H···O contacts connecting the chains into layers are shown as green dashed lines. Colour code: S, yellow; O, red; N, blue; C, grey; and H, green.

## supplementary crystallographic information

### Comment

1,3-Oxazolidin-2-ones have found use initially as chiral auxiliaries in organic synthesis (Evans *et al.*, 1981; Ager *et al.*, 1996, 1997; Hintermann & Seebach 1998) and more recently in various biological applications, *e.g.*, as a class of synthetic antibacterial agents with potent activity against clinically important susceptible and resistant Gram-positive and anaerobic pathogens (Poce *et al.*, 2008; Brickner *et al.*, 2008; Means *et al.*, 2006; Kaiser *et al.*, 2007; Clemmet & Markham, 2000; Ebner *et al.*, 2008), as interneuron blocking agents or depressants of central synaptic transmission, muscle relaxants, anticonvulsants, and tranquilizers (Negwer & Scharnow, 2007), and as potent and selective monoamine oxidase type A (MAO) inhibitors (Mai *et al.*, 2003). The syntheses of 1,3-oxazolidin-2-ones have been variously reported (Ochoa-Terán & Rivero, 2008; Zappia *et al.*, 2007).

The oxazolidin ring in (I), Fig. 1, is essentially planar with the maximum deviations of 0.036 (5) Å for atom C5 and -0.040 (4) Å for atom N1. The O5 atom lies 0.089 (3) Å out of the plane in the direction of the C2 atom, and the C6 atom is below the plane. The C2–C3–C5–C6 torsion angle of 124.6 (4) ° shows a significant twist consistent with the methanesulfonate residue being splayed out from the rest of the molecule. The methanesulfonate-methyl group is oriented towards the ring-O4 atom.

Supramolecular chains are formed in the crystal structure of (I) along the *b* direction. These are sustained by N—H···O hydrogen bonds where the oxygen acceptor is an sulfur-bound oxo group, Fig. 2 and Table 1. Three close *C*–*H*···*O*-carbonyl contacts, Table 1, provide additional stability to the chain. Chains are linked into supramolecular arrays in the *bc* plane *via* weaker C—H···O contacts and these stack along the *a* axis, Fig. 3 and Table 1.

### Experimental

A solution of (4*S*,5*R*)-4-benzyl-5-(hydroxymethyl)-1,3-oxazolidin-2-one (1.036 g, 5 mmol) and methanesulfonyl chloride (0.75 ml, 10 mmol) in triethylamine (10 ml) was stirred at room temperature for 2 h, HCl (20 ml, 15%) was added and the mixture was extracted with dichloromethane (5 *x* 20 ml). The combined organic layers were washed with brine, dried over anhydrous Na_2_SO_4_ and evaporated, giving (I) as a brown solid (0.62 g, 44%) which was recrystallized from aqueous ethanol. ^1^H-NMR (MeOD, 400 MHz): δ 7.30 (m, 5H, Ph), 4.57 (m, 1H, CHO), 4.23 (dd, 1H, ^1^J = 11.6; ^2^J = 3.0 Hz, CH_2_O), 4.11 (dd, 1H, ^1^J = 11.6; ^2^J = 4.9 Hz, CH_2_O), 4.00 (m, 1H, CHN), 3.05 (s, 3H, CH_3_), 2.92 (m, 2H, CH_2_Ph) p.p.m.; NH not obs. ^13^C-NMR (MeOD, 100 MHz): 160.4 (C═O), 137.1–128.2 (Ph), 79.5 (CHO), 70.4 (CH2O), 56.3 (CHN), 41.7 (CH_2_Ph), 37.4 (CH_3_) p.p.m. MS (m/*z*): MH^+^ 286.2.

### Refinement

The C-bound H atoms were geometrically placed (C–H = 0.95–1.00 Å) and refined as riding with *U_iso_*(H) = 1.2–1.5*U_eq_*(C). The methyl H atoms were rotated to fit the electron density. The N–H atom was located in a difference map and refined with the distance restraint N–H = 0.88±0.01 and with *U_iso_*(H) = 1.2*U_eq_*(N). The structure was refined as a racemic twin precluding the determination of the absolute structure.

### Crystal data

**Table:**

C_12_H_15_NO_5_S	*F*(000) = 300
*M**_r_* = 285.31	*D*_x_ = 1.464 Mg m^−^^3^
Monoclinic, *P*2_1_	Mo *K*α radiation, λ = 0.71073 Å
Hall symbol: P 2yb	Cell parameters from 7909 reflections
*a* = 8.7332 (5) Å	θ = 2.9–27.5°
*b* = 5.8757 (3) Å	µ = 0.27 mm^−^^1^
*c* = 12.9650 (7) Å	*T* = 120 K
β = 103.317 (3)°	Plate, colourless
*V* = 647.39 (6) Å^3^	0.26 × 0.08 × 0.02 mm
*Z* = 2	

### Data collection

**Table:**

Nonius KappaCCD area-detector diffractometer	2587 independent reflections
Radiation source: Enraf Nonius FR591 rotating anode	2266 reflections with *I* > 2σ(*I*)
10 cm confocal mirrors	*R*_int_ = 0.059
Detector resolution: 9.091 pixels mm^-1^	θ_max_ = 26.5°, θ_min_ = 3.2°
φ and ω scans	*h* = −8→10
Absorption correction: multi-scan (*SADABS*; Sheldrick, 2007)	*k* = −7→7
*T*_min_ = 0.700, *T*_max_ = 1.000	*l* = −16→14
6748 measured reflections	

### Refinement

**Table:**

Refinement on *F*^2^	Primary atom site location: structure-invariant direct methods
Least-squares matrix: full	Secondary atom site location: difference Fourier map
*R*[*F*^2^ > 2σ(*F*^2^)] = 0.057	Hydrogen site location: inferred from neighbouring sites
*wR*(*F*^2^) = 0.123	H atoms treated by a mixture of independent and constrained refinement
*S* = 1.11	*w* = 1/[σ^2^(*F*_o_^2^) + 1.5106*P*] where *P* = (*F*_o_^2^ + 2*F*_c_^2^)/3
2587 reflections	(Δ/σ)_max_ < 0.001
177 parameters	Δρ_max_ = 0.33 e Å^−^^3^
2 restraints	Δρ_min_ = −0.36 e Å^−^^3^

### Special details

**Table:**

Geometry. All s.u.'s (except the s.u. in the dihedral angle between two l.s. planes) are estimated using the full covariance matrix. The cell s.u.'s are taken into account individually in the estimation of s.u.'s in distances, angles and torsion angles; correlations between s.u.'s in cell parameters are only used when they are defined by crystal symmetry. An approximate (isotropic) treatment of cell s.u.'s is used for estimating s.u.'s involving l.s. planes.
Refinement. Refinement of *F*^2^ against ALL reflections. The weighted *R*-factor *wR* and goodness of fit *S* are based on *F*^2^, conventional *R*-factors *R* are based on *F*, with *F* set to zero for negative *F*^2^. The threshold expression of *F*^2^ > 2σ(*F*^2^) is used only for calculating *R*-factors(gt) *etc*. and is not relevant to the choice of reflections for refinement. *R*-factors based on *F*^2^ are statistically about twice as large as those based on *F*, and *R*- factors based on ALL data will be even larger.

### Fractional atomic coordinates and isotropic or equivalent isotropic displacement parameters (Å^2^)

**Table:**

	*x*	*y*	*z*	*U*_iso_*/*U*_eq_	
S1	0.44667 (11)	0.00547 (18)	0.67229 (8)	0.0215 (2)	
O1	0.6105 (3)	0.0327 (6)	0.7186 (2)	0.0284 (7)	
O2	0.3629 (4)	−0.1750 (5)	0.7089 (2)	0.0283 (8)	
O3	0.4391 (3)	−0.0275 (5)	0.5504 (2)	0.0210 (7)	
O4	0.2132 (3)	0.2865 (5)	0.4405 (2)	0.0222 (7)	
O5	0.0104 (3)	0.5267 (5)	0.4294 (2)	0.0233 (6)	
N1	−0.0074 (4)	0.2084 (6)	0.3228 (3)	0.0223 (8)	
H1N	−0.1104 (13)	0.208 (8)	0.305 (4)	0.027*	
C1	0.3493 (6)	0.2628 (8)	0.6800 (4)	0.0278 (11)	
H1A	0.3625	0.3063	0.7545	0.042*	
H1B	0.2370	0.2448	0.6473	0.042*	
H1C	0.3937	0.3815	0.6425	0.042*	
C2	0.2907 (5)	−0.1031 (7)	0.4793 (3)	0.0207 (9)	
H2A	0.2078	−0.1193	0.5196	0.025*	
H2B	0.3058	−0.2524	0.4478	0.025*	
C3	0.2425 (5)	0.0726 (7)	0.3934 (3)	0.0208 (9)	
H3	0.3280	0.0922	0.3544	0.025*	
C4	0.0626 (5)	0.3548 (7)	0.3990 (3)	0.0209 (9)	
C5	0.0868 (4)	0.0065 (9)	0.3147 (3)	0.0198 (8)	
H5	0.0396	−0.1304	0.3410	0.024*	
C6	0.1067 (5)	−0.0349 (8)	0.2020 (3)	0.0246 (10)	
H6A	0.1506	0.1036	0.1763	0.030*	
H6B	0.1824	−0.1607	0.2030	0.030*	
C7	−0.0472 (5)	−0.0941 (8)	0.1270 (3)	0.0229 (9)	
C8	−0.1231 (5)	0.0627 (8)	0.0526 (3)	0.0289 (11)	
H8	−0.0773	0.2083	0.0492	0.035*	
C9	−0.2646 (5)	0.0105 (11)	−0.0168 (3)	0.0333 (10)	
H9	−0.3146	0.1199	−0.0674	0.040*	
C10	−0.3333 (6)	−0.1999 (9)	−0.0126 (4)	0.0326 (11)	
H10	−0.4308	−0.2360	−0.0597	0.039*	
C11	−0.2577 (6)	−0.3580 (8)	0.0615 (4)	0.0301 (11)	
H11	−0.3037	−0.5033	0.0653	0.036*	
C12	−0.1161 (5)	−0.3054 (8)	0.1297 (4)	0.0267 (10)	
H12	−0.0651	−0.4160	0.1792	0.032*	

### Atomic displacement parameters (Å^2^)

**Table:**

	*U*^11^	*U*^22^	*U*^33^	*U*^12^	*U*^13^	*U*^23^
S1	0.0215 (5)	0.0219 (5)	0.0206 (5)	−0.0014 (5)	0.0037 (4)	−0.0012 (5)
O1	0.0221 (14)	0.036 (2)	0.0237 (15)	−0.0046 (16)	−0.0008 (11)	−0.0039 (15)
O2	0.0333 (19)	0.0263 (18)	0.0235 (17)	−0.0045 (15)	0.0030 (14)	0.0029 (14)
O3	0.0161 (13)	0.0277 (18)	0.0178 (13)	−0.0015 (13)	0.0007 (10)	−0.0043 (13)
O4	0.0176 (15)	0.0207 (15)	0.0260 (16)	0.0016 (12)	0.0001 (12)	−0.0014 (13)
O5	0.0240 (14)	0.0174 (16)	0.0294 (15)	0.0013 (14)	0.0082 (12)	0.0000 (14)
N1	0.0172 (18)	0.026 (2)	0.024 (2)	0.0008 (15)	0.0046 (15)	−0.0014 (15)
C1	0.032 (3)	0.021 (2)	0.031 (3)	0.000 (2)	0.009 (2)	−0.005 (2)
C2	0.020 (2)	0.022 (2)	0.019 (2)	0.0003 (17)	0.0018 (17)	−0.0022 (17)
C3	0.020 (2)	0.014 (2)	0.028 (2)	−0.0019 (15)	0.0052 (17)	−0.0025 (16)
C4	0.022 (2)	0.021 (2)	0.021 (2)	−0.0033 (18)	0.0066 (17)	0.0057 (17)
C5	0.0181 (17)	0.0195 (19)	0.0217 (19)	0.001 (2)	0.0041 (14)	0.006 (2)
C6	0.022 (2)	0.026 (3)	0.025 (2)	0.0009 (18)	0.0060 (17)	0.0007 (18)
C7	0.027 (2)	0.026 (2)	0.017 (2)	0.0023 (18)	0.0080 (18)	−0.0022 (17)
C8	0.035 (2)	0.029 (3)	0.024 (2)	−0.001 (2)	0.0093 (19)	0.0006 (18)
C9	0.041 (2)	0.035 (3)	0.020 (2)	0.004 (3)	0.0000 (18)	−0.003 (3)
C10	0.027 (2)	0.043 (3)	0.027 (3)	0.003 (2)	0.003 (2)	−0.007 (2)
C11	0.033 (3)	0.024 (3)	0.034 (3)	−0.005 (2)	0.008 (2)	−0.003 (2)
C12	0.029 (2)	0.028 (2)	0.022 (2)	−0.001 (2)	0.0024 (18)	0.0016 (19)

### Geometric parameters (Å, °)

**Table:**

S1—O1	1.427 (3)	C3—H3	1.0000
S1—O2	1.430 (3)	C5—C6	1.529 (5)
S1—O3	1.578 (3)	C5—H5	1.0000
S1—C1	1.749 (5)	C6—C7	1.507 (6)
O3—C2	1.475 (5)	C6—H6A	0.9900
O4—C4	1.362 (5)	C6—H6B	0.9900
O4—C3	1.445 (5)	C7—C12	1.383 (6)
O5—C4	1.210 (5)	C7—C8	1.387 (6)
N1—C4	1.346 (6)	C8—C9	1.385 (6)
N1—C5	1.462 (6)	C8—H8	0.9500
N1—H1N	0.875 (10)	C9—C10	1.381 (8)
C1—H1A	0.9800	C9—H9	0.9500
C1—H1B	0.9800	C10—C11	1.390 (7)
C1—H1C	0.9800	C10—H10	0.9500
C2—C3	1.506 (5)	C11—C12	1.379 (7)
C2—H2A	0.9900	C11—H11	0.9500
C2—H2B	0.9900	C12—H12	0.9500
C3—C5	1.550 (5)		
			
O1—S1—O2	118.9 (2)	N1—C4—O4	109.6 (4)
O1—S1—O3	103.99 (16)	N1—C5—C6	112.8 (3)
O2—S1—O3	109.53 (18)	N1—C5—C3	99.9 (4)
O1—S1—C1	109.4 (2)	C6—C5—C3	113.2 (3)
O2—S1—C1	109.2 (2)	N1—C5—H5	110.2
O3—S1—C1	104.8 (2)	C6—C5—H5	110.2
C2—O3—S1	119.4 (2)	C3—C5—H5	110.2
C4—O4—C3	109.8 (3)	C7—C6—C5	111.9 (3)
C4—N1—C5	113.8 (4)	C7—C6—H6A	109.2
C4—N1—H1N	117 (3)	C5—C6—H6A	109.2
C5—N1—H1N	123 (3)	C7—C6—H6B	109.2
S1—C1—H1A	109.5	C5—C6—H6B	109.2
S1—C1—H1B	109.5	H6A—C6—H6B	107.9
H1A—C1—H1B	109.5	C12—C7—C8	118.2 (4)
S1—C1—H1C	109.5	C12—C7—C6	121.3 (4)
H1A—C1—H1C	109.5	C8—C7—C6	120.5 (4)
H1B—C1—H1C	109.5	C9—C8—C7	121.1 (5)
O3—C2—C3	108.1 (3)	C9—C8—H8	119.4
O3—C2—H2A	110.1	C7—C8—H8	119.4
C3—C2—H2A	110.1	C10—C9—C8	120.2 (5)
O3—C2—H2B	110.1	C10—C9—H9	119.9
C3—C2—H2B	110.1	C8—C9—H9	119.9
H2A—C2—H2B	108.4	C9—C10—C11	118.9 (4)
O4—C3—C2	109.3 (3)	C9—C10—H10	120.5
O4—C3—C5	106.4 (3)	C11—C10—H10	120.5
C2—C3—C5	111.6 (3)	C12—C11—C10	120.4 (5)
O4—C3—H3	109.8	C12—C11—H11	119.8
C2—C3—H3	109.8	C10—C11—H11	119.8
C5—C3—H3	109.8	C11—C12—C7	121.1 (4)
O5—C4—N1	129.1 (4)	C11—C12—H12	119.5
O5—C4—O4	121.3 (4)	C7—C12—H12	119.5
			
O1—S1—O3—C2	168.8 (3)	C2—C3—C5—N1	123.5 (4)
O2—S1—O3—C2	40.7 (4)	O4—C3—C5—C6	124.6 (4)
C1—S1—O3—C2	−76.4 (3)	C2—C3—C5—C6	−116.3 (4)
S1—O3—C2—C3	122.4 (3)	N1—C5—C6—C7	−66.3 (5)
C4—O4—C3—C2	−121.3 (4)	C3—C5—C6—C7	−178.9 (4)
C4—O4—C3—C5	−0.7 (4)	C5—C6—C7—C12	−73.9 (5)
O3—C2—C3—O4	−62.6 (4)	C5—C6—C7—C8	106.5 (5)
O3—C2—C3—C5	180.0 (3)	C12—C7—C8—C9	0.4 (6)
C5—N1—C4—O5	−174.4 (4)	C6—C7—C8—C9	−180.0 (4)
C5—N1—C4—O4	7.3 (5)	C7—C8—C9—C10	0.3 (7)
C3—O4—C4—O5	177.7 (4)	C8—C9—C10—C11	−0.5 (7)
C3—O4—C4—N1	−3.8 (5)	C9—C10—C11—C12	−0.1 (7)
C4—N1—C5—C6	−127.6 (4)	C10—C11—C12—C7	0.9 (7)
C4—N1—C5—C3	−7.1 (4)	C8—C7—C12—C11	−1.0 (7)
O4—C3—C5—N1	4.4 (4)	C6—C7—C12—C11	179.4 (4)

### Hydrogen-bond geometry (Å, °)

**Table:**

*D*—H···*A*	*D*—H	H···*A*	*D*···*A*	*D*—H···*A*
N1—H1N···O2^i^	0.88 (2)	2.28 (2)	3.113 (5)	160 (4)
C1—H1B···O5^ii^	0.98	2.51	3.428 (6)	155
C2—H2A···O5^ii^	0.99	2.32	3.214 (5)	150
C5—H5···O5^iii^	1.00	2.36	3.326 (6)	162
C6—H6B···O1^iv^	0.99	2.59	3.528 (6)	158

Symmetry codes: (i) −*x*, *y*+1/2, −*z*+1; (ii) −*x*, *y*−1/2, −*z*+1; (iii) *x*, *y*−1, *z*; (iv) −*x*+1, *y*−1/2, −*z*+1.

## References

[bb1] Ager, D. J., Prakash, I. & Schaad, D. R. (1996). *Chem. Rev.* **96**, 835–876. 10.1021/cr9500038 11848773

[bb2] Ager, D. J., Prakash, I. & Schaad, D. R. (1997). *Aldrichim. Acta*, **30**, 3–11.

[bb3] Brandenburg, K. (2006). *DIAMOND* Crystal Impact GbR, Bonn, Germany.

[bb4] Brickner, S. J., Barbachyn, M. R., Hutchinson, D. K. & Manninen, P. R. (2008). *J. Med. Chem.* **51**, 1981–1990. 10.1021/jm800038g 18338841

[bb5] Clemmet, D. & Markham, A. (2000). *Drugs*, **59**, 815–827. 10.2165/00003495-200059040-00007 10804037

[bb6] Ebner, D. C., Culhane, J. C., Winkelman, T. N., Haustein, M. D., Ditty, J. L. & Ippoliti, J. T. (2008). *Bioorg. Med. Chem.* **16**, 2651–2656. 10.1016/j.bmc.2007.11.040 18077175

[bb7] Evans, D. A., Bartroli, J. & Shih, T. L. (1981). *J. Am. Chem. Soc.* **103**, 2127–2129.

[bb8] Farrugia, L. J. (1997). *J. Appl. Cryst.* **30**, 565.

[bb9] Hintermann, T. & Seebach, D. (1998). *Helv. Chim. Acta*, **81**, 2093–2126.

[bb10] Hooft, R. W. W. (1998). *COLLECT* Nonius BV, Delft, The Netherlands.

[bb11] Kaiser, C., Cunico, W., Pinheiro, A. C., de Oliveira, A. G. & Souza, M. V. N. (2007). *Rev. Brás. Farm.* **88**, 83–88.

[bb12] Mai, A., Artico, M., Esposito, M., Ragno, R., Sbardella, G. & Massa, S. (2003). *Il Farmaco*, **58**, 231–241. 10.1016/S0014-827X(03)00016-8 12620419

[bb13] Means, J. A., Katz, S., Nayek, A., Anupm, R., Hines, J. V. & Bergmeier, S. C. (2006). *Bioorg. Med. Chem. Lett.* **16**, 3600–3604. 10.1016/j.bmcl.2006.03.068 16603349

[bb14] Negwer, M. & Scharnow, H. G. (2007). *Organic Chemical Drugs and Their Synonyms*, 9th ed. Weinheim, Germany: Wiley–VCH.

[bb15] Ochoa-Terán, A. & Rivero, I. A. (2008). *Arkivoc*, pp. 330–343.

[bb16] Otwinowski, Z. & Minor, W. (1997). *Methods in Enzymology*, Vol. 276, *Macromolecular Crystallography*, Part A, edited by C. W. Carter Jr & R. M. Sweet, pp. 307–326. New York: Academic Press.

[bb17] Poce, G., Zappia, G., Porretta, G. C., Botta, B. & Biava, M. (2008). *Exp. Opin. Ther. Patents*, **18**, 97–121.

[bb18] Sheldrick, G. M. (2007). *SADABS* Bruker AXS Inc., Madison, Wisconsin, USA.

[bb19] Sheldrick, G. M. (2008). *Acta Cryst.* A**64**, 112–122. 10.1107/S0108767307043930 18156677

[bb20] Westrip, S. P. (2010). *publCIF* In preparation.

[bb21] Zappia, G., Gacs-Baitz, E., Delle Monache, G., Misiti, D., Nevola, L. & Botta, B. (2007). *Curr. Org. Synth.* **4**, 81–135.

